# Translation, cultural adaption and content validity evaluation of the Occupational Balance Questionnaire into German—a cross-sectional study in Austria, Germany, and Switzerland

**DOI:** 10.3389/fpubh.2026.1753366

**Published:** 2026-02-16

**Authors:** Lisa Dorfer, Valentin Ritschl, Patric Duletzki, Alexandra Radek, Carita Håkansson, Petra Wagman, Margaret Renn Andrews, Erika Mosor, Tanja Alexandra Stamm

**Affiliations:** 1 Institute for Outcomes Research, Centre for Medical Data Science, Medical University of Vienna, Vienna, Austria; 2 Ludwig Boltzmann Institute for Arthritis and Rehabilitation, Vienna, Austria; 3 Recura Akademie für Sozial- und Gesundheitsberufe, Potsdam, Germany; 4 Independent Practitioner, Uelzen, Germany; 5 Division of Occupational and Environmental Medicine, Lund University, Lund, Sweden; 6 School of Health and Welfare, Jönköping University, Jönköping, Sweden

**Keywords:** Occupational Balance, Occupational Balance Questionnaire (OBQ11), patient reported outcome measure (PROM), translation, work life balance

## Abstract

**Background:**

Extending the concept of work-life balance and recognising that many people’s lives cannot easily be divided into work and non-work life, Occupational Balance enables the exploration of a person’s satisfaction with the balance and variance of occupations in daily life at most contemporary levels.

**Objective:**

To effectively measure Occupational Balance in the German-speaking population, this study developed a German version of the Occupational Balance Questionnaire (OBQ11-G) by conducting a cross-sectional study involving translation, cultural adaptation and content validity evaluation.

**Methods:**

A preliminary German version of the OBQ11 was developed based on Beaton’s guideline and then finalised after an online focus group (*n* = 7 occupational therapists; 5 women [71%]) to reach consensus on the relevance, comprehensibility and overall comprehensiveness of the items. We applied the framework method to analyse the focus group’s qualitative data and descriptive statistics to report the quantitative data.

**Results:**

Overall, the experts rated all questionnaire items as “relevant” to “very relevant”, but eight of the eleven items were slightly adapted to improve comprehensibility, due to inconsistencies in terminology. Experts did not agree on whether the questionnaire fully covered the concept of Occupational Balance, with 57% of respondents agreeing and 43% disagreeing, suggesting that additional assessments should be applied when measuring Occupational Balance.

**Conclusion:**

The German version of the OBQ11 has now been thoroughly translated, and further studies on its psychometric properties are needed to enhance its validity.

## 1 Introduction

Occupations are activities people engage in to fulfil specific purposes such as self-care, productivity and leisure (1). Engagement in occupations can be health-promoting due to the individual’s direct physical, cognitive, and social benefits (2) and influences life satisfaction and well-being in general. However, the loss of engagement, for example, in work-related activities when unemployed, may be associated with poorer health outcomes (3). Another important aspect when considering a person’s health and well-being in relation to occupations is how people experience a balance or imbalance of their mix of activities in daily life (4). In this context, the concept of work-life balance has arguably become the most popular in public discourse (5). It refers to the relationship between the work and non-work aspects of a person’s life, where achieving a satisfactory work-life balance is usually understood as cutting back on one side to have more time for the other (5). However, the concept of work-life balance must be viewed critically, as it has a limited idea of what “life” means. A concept being based on a traditional work model (8 h work, sleep and leisure each) does not take into account changes in gender roles and recent developments in work and employment relationships (6), time for self-care, caring responsibilities and more. While work-life balance focuses specifically on harmonising work demands with personal life, Occupational Balance is a broader concept that encompasses all daily activities.

Occupational Balance emphasises that one’s life cannot be simply divided into work and non-work life. It describes the subjective perception that “the right number of occupations/activities and the right variety between them” (7) has a positive influence on a person’s health and well-being (8). Occupational Balance is a popular concept among occupational therapists and scientists and is associated with health promotion in several populations with chronic conditions, including people with inflammatory arthritis (9), fibromyalgia (10), and acquired brain injury (11). A study by Dür and colleagues (12) found preliminary evidence for biological links between occupation and health by showing an association between Occupational Balance, functioning, cytokines and c-reactive protein (CRP). Despite these consistent results, the concept of Occupational Balance is still not uniformly used, defined, or understood, making it difficult to measure it in a standardised way and compare intervention or study results. Therefore, Wagman and colleagues (7) conducted a comprehensive concept analysis to reach a consensus on the Occupational Balance concept and developed the Occupational Balance Questionnaire (OBQ11). Within the last years, the OBQ11 was revised and then validated in many studies (13, 14).

The OBQ11 focuses on the variation in occupational patterns, the time spent in each occupation, and the mix of activities and their individually perceived meaningfulness (15). The eleven items have four response alternatives with verbal descriptions. Participants rate their level of agreement with various assumptions about their Occupational Balance (0 = strongly disagree, 1 = disagree, 2 = agree, 3 = strongly agree). Item scores are summed into total scores ranging from 0 to 33. The OBQ11 has not been available in German. The aim of this study was therefore to translate and adapt the questionnaire cross-culturally, providing a German version of the OBQ11 and a preliminary content validity assessment for use in further psychometric analysis.

## 2 Methods

### 2.1 Study design

The authors conducted a cross-sectional study to translate and cross-culturally adapt the OBQ11 into German and evaluate its content validity. The COSMIN Study Design checklist for patient-reported outcome measurement instruments was used as reporting guideline (16).

### 2.2 Participants

Individuals with knowledge of German and English and who were familiar with the Occupational Balance concept were selected to translate the OBQ11. For the focus group, occupational therapists from Austria, Germany and Switzerland who were familiar with the Occupational Balance concept were selected. Other inclusion criteria were at least one year of experience in occupational therapy and German as a first language. Sampling was purposeful. According to Austrian law, expert studies do not need to be submitted to the ethics committee. All experts provided verbal consent at the beginning of the interviews, having been informed in detail about the study.

### 2.3 Procedures

The authors obtained permission from the original OBQ11 development team to translate the instrument, and accessibility was granted upon request. A rigorous translation process was followed to ensure cultural sensitivity, adhering to the guideline by Beaton et al. (17), prioritizing semantic, idiomatic and conceptual equivalence between the source and target languages. This involved the following steps.

#### 2.3.1 Initial translation

One translator from each German-speaking (Austria, Germany, Switzerland) country (with a language level of at least C1) translated the assessment from English into German. The translations were as close to the original as possible considering cultural and linguistic differences. The authors then produced an initial German translation based on the three individual translations.

#### 2.3.2 Back translation

Three bilingual translators, unfamiliar with the original instrument, translated the harmonised translation back into the source language (English) to enable the authors of this paper to identify any inconsistencies or misunderstandings.

#### 2.3.3 Comparison and alignment

The three back-translated versions were then compared with the original instrument. Any discrepancies were reconciled through discussion and revision to ensure conceptual equivalence. A preliminary German version of the questionnaire was then created for further review.

#### 2.3.4 Expert panel review

In an online focus group, the seven experts (five female and two male) assessed the translation for conceptual equivalence, cultural relevance, and potential distortions. They were all occupational therapists, and their different professional specialisations meant that they formed a heterogeneous group of experts. While all of them were familiar with the concept of Occupational Balance, a requirement for participation in the focus group, two of them worked in neurology, two in psychiatry, one in hand therapy, and two worked as research assistants based in Austria, Germany, and Switzerland. They all had at least one year of work experience, and their mean age was 30.57 (±9.61). Additionally, validity was assessed in line with cross-cultural adaptation practices for health status screening instruments (18). Content validity, crucial for robust instrument development (19), was also evaluated as part of this focus group. Further details on this process are provided in the data collection and analysis section.

#### 2.3.5 Finalisation

The final translated instrument was created after revisions and improvements based on the expert panel’s feedback from the focus group. A comprehensive record documenting the entire process, including revisions and decisions made by the expert panel, is available on request.

### 2.4 Data collection

The focus group was conducted online via Webex^1^ due to the geographical distribution of the participants and was led by one of the authors (PD). Supported by two other authors, AR and LD where responsible for observing the group and recording data. They also wrote notes relating to descriptive observations of the group interaction and discussion topics.

At the beginning of the focus group, the moderator explained the procedure and the concept of Occupational Balance to all participants according to the definition of Wagman’s concept analysis (7). Every item of the OBQ11 was presented on a slide in English and German. After the items were read out, the participants were asked for open comments and thoughts on it, especially its translation. For the structured assessment of content validity, participants were asked to rate the relevance and comprehensibility of each item and the overall comprehensibility of the questionnaire. The online tool Poll Everywhere^2^ allowed to anonymously rate the comprehensibility (1 = not clear; 2 = should be revised to provide more clarity; 3 = is very clear), relevance (1 = not relevant; 2 = somewhat relevant; 3 = fairly relevant; 4 = very relevant) and comprehensiveness of the overall questionnaire (yes, no) (20). Therefore, it was possible to collect qualitative information about the thoughts of the individual participant in addition to an overall quantitative assessment.

The focus group was recorded and transcribed verbatim using an online tool (see text footnote 1).

### 2.5 Data analysis

The researchers used the matrix-based Framework method to analyse the qualitative data (21, 22). They entered the transcripts of the focus groups into a series of grids. Each row represented an individual participant, and each column represented an area of enquiry. The areas of enquiry, in this case, each item of the OBQ11, were in the same order as in the original questionnaire to allow the authors to systematically review the collected data (23). Initially, AR and LD, completed the matrices independently. Afterward, their versions were compared and consolidated into a single file with the input of PD in case of inconsistencies. This approach was intended to ensure researcher triangulation.

After completing the matrices with text and analysing the qualitative data, statistical analysis for descriptive statistics was conducted to evaluate the scores of comprehensibility and relevance of each item. Relative and absolute frequencies were calculated. As for comprehensibility, further review of the individual items was considered necessary if less than 70% of the participants scored 3 (meaning the item *is very clear*). Concerning relevance, discussions were deepened when less than 70% of the experts voted for 3 (*fairly relevant*) or 4 (*very relevant*). The 70% agreement threshold used in this analysis is derived from the Delphi study rule, where 70% is a common benchmark for reaching consensus (24).

## 3 Results

A preliminary version of the OBQ11-G was created following the initial translation, back translation, comparison and alignment steps according to Beaton’s guidelines (17).

The focus group consisted of seven occupational therapists who are experts in the concept of Occupational Balance. As well as supporting the translation and cross-cultural adaptation, the focus group was primarily responsible for evaluating the content validity of the OBQ11-G. The results of the structured assessment of the content validity, relevance, and comprehensibility of the individual items and the questionnaire’s overall comprehensiveness can be found in Tables 1, 2. Further review of an item was considered necessary for comprehensibility if fewer than 70% of participants voted 3 (marked in red), a common threshold for consensus. No further discussion was necessary regarding relevance, as more than 70% of participants voted 3 or above for each item (marked in green). Overall, the experts rated all items as ‘relevant’ to ‘very relevant’, but eight of the 11 items were adapted in terms of comprehensibility, partly due to inconsistencies in terminology.

**Table 1:** Relevance of the OBQ11 items.

	Questionnaire titel	Item1	Item2	Item3	Item4	Item5	Item6	Item 7	Item8	Item 9	Item 10	Item 11
1	0%	0%	0%	0%	0%	0%	0%	0%	14%	29%	0%	0%
2	15%	0%	14%	29%	0%	0%	0%	0%	0%	14%	14%	29%
3	57%	33%	57%	29%	0%	57%	9%	29%	14%	14%	29%	14%
4	29%	67%	29%	43%	100%	43%	91%	71%	71%	43%	57%	57%

1 = not relevant; 2 = somewhat relevant; 3 = fairly relevant; 4 = very relevant. Green indicates that no item changes were necessary.

**Table 2:** Comprehensibility of the OBQ11 items.

	Titel	Item1	Item2	Item3	Item4	Item5	Item6	Item 7	Item8	Item 9	Item 10	Item 11
1	0%	0%	14%	0%	0%	0%	0%	0%	0%	0%	0%	0%
2	14%	100%	71%	57%	71%	29%	57%	86%	71%	29%	29%	71%
3	86%	0%	14%	43%	29%	71%	43%	14%	29%	71%	71%	29%

1 = not clear; 2 = should be revised to provide more clarity; 3 = is very clear.Green indicates that no item changes were necessary, whereas red indicates that changes were necessary.

As indicated in Table 3, adjustments to the items were mainly required due to inconsistencies in occupational therapy terminology in German. However, the meanings of the terms, as well as their different interpretations and uses in practice, were also discussed. In addition to the wording adjustments shown in Table 3, several respondents emphasised that the OBQ11 should not be used solely to assess Occupational Balance as this may not provide a complete picture. For example, it may lack information about an individual’s occupational patterns that would be necessary to help them improve their Occupational Balance. This aligns with the quantitative findings on the comprehensiveness of the questionnaire: 57% of respondents found it comprehensive, while 43% disagreed (*n* = 7). One participant stated, “If I were to complete this questionnaire with a patient, I would require a second assessment. It is not enough to just collect this information. I think it would be important to be more specific. The questionnaire asks about quantity (of activities), but it would be easier to understand if it were based on a weekly schedule filled out by the patient”. Meanwhile, a participant who rated the assessment as comprehensive stated, “I would use this questionnaire directly for the initial consultation. It’s nice and short, gives a quick first impression, is practical to use and leads me to where I want to go in more detail and see where I can draw on my repertoire”.

**Table 3:** Results of the translational and adaptation process of the German OBQ11.

	OBQ11 items (short form)	OBQ11-German (preliminary)	OBQ11-German (final)
Title	Occupational Balance Questionnaire (OBQ11)	Fragebogen zur Betätigungsbalance Occupational Balance Questionnaire (OBQ11-GERMAN)	Fragebogen zur Betätigungsbalance Occupational Balance Questionnaire (OBQ11-GERMAN)
Item 1	Having just enough to do during a regular week	In einer normalen Woche habe ich das Gefühl, dass die Anzahl der Dinge, die ich tue, genau richtig ist.	Ich habe das Gefühl, dass es genau die richtige Menge an Aktivitäten gibt, die in einer gewöhnlichen Woche zu tun sind.
Item 2	Balance between doing things for others and for oneself	Es besteht eine Balance zwischen Dingen, die ich für mich tue, und Dingen, die ich für andere tue.	Es besteht eine Balance zwischen Aktivitäten, die ich für mich selbst tue, und Aktivitäten, die ich für andere tue.
Item 3	Perceiving one’s occupations as meaningful	Ich achte darauf, Dinge zu tun, die ich wirklich tun möchte.	Ich achte darauf, Aktivitäten zu tun, die ich wirklich tun möchte.
Item 4	Balance between work, home, family, leisure, rest, and sleep	Ich schaffe eine Balance zwischen den verschiedenen Aktivitäten in meinem Leben, z. B. Arbeit, Haushalt, Freizeit, Ruhe und Schlaf.	Ich schaffe eine Balance zwischen den verschiedenen Aktivitäten in meinem Leben, z. B. Arbeit, Haushalt, Freizeit, Ruhe und Schlaf.
Item 6	Balance between physical, social, mental, and restful occupations	Ich habe eine Balance zwischen meinen körperlichen, sozialen, geistigen und erholsamen Aktivitäten.	Ich habe eine Balance zwischen meinen körperlichen, sozialen, kognitiven und erholsamen Aktivitäten.
Item 7	Satisfaction with how time is spent in everyday life	Mit der Menge an Zeit, die ich mit meinen verschiedenen täglichen Aktivitäten verbringe, bin ich zufrieden.	Ich bin zufrieden mit der Zeit, die ich für meine verschiedenen täglichen Aktivitäten aufwende.
Item 8	Satisfaction with the number of activities during a regular week	Ich bin zufrieden mit der Anzahl an Aktivitäten, an denen ich in einer normalen Woche teilnehme.	Ich bin zufrieden mit der Anzahl der Aktivitäten, an denen ich in einer gewöhnlichen Woche teilnehme.
Item 11	Satisfaction with time spent in rest, recovery, and sleep	Mit der Menge an Zeit, die ich mit Entspannung, Erholung und Schlaf verbringe, bin ich zufrieden.	Ich bin zufrieden mit der Zeit, die ich mit Entspannung, Erholung und Schlaf verbringe.

After incorporating the necessary revisions and conducting a final review, the final version of the German OBQ11 (OBQ11-G) was produced.

## 4 Discussion

In this paper, we present the German-language version of the OBQ11, referred to as OBQ11-G. This version is designed to assess Occupational Balance in German-speaking countries, including Austria, Germany, and Switzerland. Following a rigorous translation and adaptation process, the OBQ11-G appears to be a thorough translation of the English OBQ11 version, however, its psychometric properties require further exploration.

In comparison to existing translation and cross-cultural adaptation studies, the OBQ11 translation from Swedish to Norwegian was relatively straightforward, as expected, given the similarity of the languages (14). The same was true of the OBQ11 translations into Arabic and Turkish (13, 25). However, the German translation posed more challenges in terms of vocabulary and the comprehensiveness of the OBQ11. The issue of the OBQ11’s comprehensiveness had not previously been reported, and the challenge posed by occupational therapy-specific vocabulary appears to be more prevalent among German-speaking occupational therapists than among those speaking Scandinavian, Turkish or Arabic.

The main reason for the need for adaptation during the translation process into German was inconsistencies in the terminology of German-speaking occupational therapists. Language differences, particularly the difficulties in non-Anglophone countries, have been mentioned in occupational therapy and science before. As early as 2006, Creek pointed out that occupational therapists struggled to agree on the meaning of key terms in their profession (26). Another terminological issue in the discipline is that most occupational therapy literature is written in English. When occupational therapists attempt to translate these texts into other languages, they often find it challenging to find suitable words to translate the key terms. Despite the efforts of the European Network of Occupational Therapy in Higher Education (ENOTHE) terminology project group (27), which aimed to create a common understanding and harmonise occupational therapy terminology to improve communication among occupational therapists in English, Dutch, French, German, Portuguese and Spanish, there are still differences in how occupational therapists refer to their work. To understand the discrepancies, further investigation of the professional language used by occupational therapists in German-speaking countries is necessary. In addition, the experts report that the limited comprehensiveness of the OBQ11 needs to be further investigated, as this may be due to a lack of consensus on the wording or interpretation of the term “Occupational Balance”.

Despite the challenges encountered in translating and adapting the OBQ11-G, the use of standardized instruments generally has several advantages, including the ability to compare a client’s condition with others, predict recovery, support treatment planning and monitoring, and facilitate communication with clients, families, health professionals, and other institutions (28). And although occupation-based instruments are used less frequently than impairment-based instruments (29), we strongly advocate for the adoption of standardized occupation-based instruments, despite terminological differences, as this could eliminate the difficulties occupational therapists face in their daily practice and promote consensus on occupational therapy-specific terms. Therefore, the adoption of the thoroughly revised OBQ11-G is recommended, especially after further psychometric evaluations.

### 4.1 Limitations

Experts did not agree on whether the questionnaire fully covered the concept of Occupational Balance, with 57% of respondents agreeing and 43% disagreeing, suggesting that additional assessments should be applied when measuring Occupational Balance. A second round consensus procedure was not conducted due to the limited availability of the experts; whether to include the items with <70% agreement should therefore be subject to further analysis. Future studies on its psychometric properties are needed to increase its validity and comprehensibility, as well as to address the issue of comprehensiveness. Furthermore, although the COSMIN guidelines were followed, the evaluation of content validity was based on a limited number of experts. Therefore, while this study provides a thoroughly translated version that follows standard guidelines, more psychometric properties need to be evaluated to provide a standardised assessment.

## 5 Conclusion

A rigorous translation process led to the creation of a German version of the OBQ11, the OBQ11-G, and further studies on its psychometric properties are needed to improve its validity and also to investigate challenges such as the lack of consensus on terminology among German-speaking occupational therapists and the limited comprehensivenss of the OBQ11-G.

## Data Availability

The datasets presented in this article are not readily available because data are available upon reasonable request. Requests to access the datasets should be directed to lisa.dorfer@meduniwien.ac.at.

## Footnotes

1
www.webex.com

2
www.polleverywhere.com

## References

[1] Townsend E. A. Polatajko H. J., and Canadian Association of Occupational Therapists. (2007). Enabling occupation II: Advancing an occupational therapy vision for health, well-being & justice through occupation. Canadian Association of Occupational Therapists. Ottawa.

[2] Leland NE Elliott SJ. Special issue on productive aging: evidence and opportunities for occupational therapy practitioners. Am J Occup Ther. (2012) 66:263–5. doi: 10.5014/ajot.2010.005165, 22549589 PMC5364020

[3] The Lancet Public Health. Public health and the workplace: a new era dawns. Lancet Public Health. (2018) 3:e508. doi: 10.1016/S2468-2667(18)30217-2 30409396

[4] Backman CL. Occupational balance: exploring the relationships among daily occupations and their influence on well-being. Can J Occup Ther. (2004) 71:202–9. doi: 10.1177/000841740407100404, 15586852

[5] Kelliher C Richardson J Boiarintseva G. All of work? All of life? Reconceptualising work-life balance for the 21st century. Hum Resour Manage J. (2019) 29:97–112. doi: 10.1111/1748-8583.12215

[6] Korunka C. Flexible working practices and approaches: Psychological and social implications. New York: Springer International Publishing (2021).

[7] Wagman P Håkansson C Björklund A. Occupational balance as used in occupational therapy: a concept analysis. Scand J Occup Ther. (2012) 19:322–7. doi: 10.3109/11038128.2011.596219, 21780985

[8] Håkansson C Milevi S Eek F Oudin A Wagman P. Occupational balance, work and life satisfaction in working cohabiting parents in Sweden. Scand J Public Health. (2019) 47:366–74. doi: 10.1177/1403494819828870, 30813858

[9] To-Miles F Håkansson C Wagman P Backman CL. Exploring the associations among occupational balance and health of adults with and without inflammatory arthritis. Arthritis Care Res (Hoboken). (2022) 74:22–30. doi: 10.1002/acr.24732, 34121370

[10] Ortiz-Rubio A Cabrera-Martos I Haro-Piedra E López-López L Rodríguez-Torres J Granados-Santiago M . Exploring perceived occupational balance in women with fibromyalgia. A descriptive study. Scand J Occup Ther. (2022) 29:395–402. doi: 10.1080/11038128.2020.1865449, 33369515

[11] Nyman A Kassberg AC Lund ML. Perceived occupational value in people with acquired brain injury. Scand J Occup Ther. (2021) 28:391–8. doi: 10.1080/11038128.2020.1791951, 32669015

[12] Dür M Steiner G Stoffer MA Fialka-Moser V Kautzky-Willer A Dejaco C . Initial evidence for the link between activities and health: associations between a balance of activities, functioning and serum levels of cytokines and C-reactive protein. Psychoneuroendocrinology. (2016) 65:138–48. doi: 10.1016/j.psyneuen.2015.12.015, 26773841

[13] Günal A Pekçetin S Demirtürk F Şenol H Håkansson C Wagman P. Validity and reliability of the Turkish occupational balance questionnaire (OBQ11-T). Scand J Occup Ther. (2020) 27:493–9. doi: 10.1080/11038128.2019.1673479, 31608736

[14] Uhrmann L Hovengen I Wagman P Håkansson C Bonsaksen T. The Norwegian occupational balance questionnaire (OBQ11-N)—development and pilot study. Scand J Occup Ther. (2019) 26:546–51. doi: 10.1080/11038128.2018.1523458, 30422027

[15] Wagman P Håkansson C. Introducing the occupational balance questionnaire (OBQ). Scand J Occup Ther. (2014) 21:227–31. doi: 10.3109/11038128.2014.900571, 24649971

[16] COSMIN. (2019). COSMIN Study Design checklist for Patient-reported outcome measurement instruments (accessed 15.11.2021). Available online at: https://www.cosmin.nl/wp-content/uploads/COSMIN-study-designing-checklist_final.pdf

[17] Beaton DE Bombardier C Guillemin F Ferraz MB. Guidelines for the process of cross-cultural adaptation of self-report measures. Spine (Phila Pa 1976). (2000) 25:3186–91. doi: 10.1097/00007632-200012150-00014, 11124735

[18] Anthoine E Moret L Regnault A Sébille V Hardouin JB. Sample size used to validate a scale: a review of publications on newly-developed patient reported outcomes measures. Health Qual Life Outcomes. (2014) 12:176. doi: 10.1186/s12955-014-0176-2 25492701 PMC4275948

[19] Brod M Tesler LE Christensen TL. Qualitative research and content validity: developing best practices based on science and experience. Qual Life Res. (2009) 18:1263–78. doi: 10.1007/s11136-009-9540-9, 19784865

[20] Zamanzadeh V Ghahramanian A Rassouli M Abbaszadeh A Alavi-Majd H Nikanfar AR. Design and implementation content validity study: development of an instrument for measuring patient-centered communication. J Caring Sci. (2015) 4:165–78. doi: 10.15171/jcs.2015.017, 26161370 PMC4484991

[21] Miles MB Huberman AM. Qualitative data analysis: An expanded sourcebook. London: Sage Publications (1994).

[22] Spencer L Ritchie J O'Connor W. Analysis: practices, principles and processes In: Ritchie J Lewis J, editors. Qualitative research practice. 1st ed. London: Sage Publications (2003). 199–218.

[23] D'ardenne J Collins D. Data Management In: Collins D, editor. Cognitive interviewing practice. London: SAGE (2015)

[24] De Meyer D Kottner J Beele H Schmitt J Lange T Van Hecke A . Delphi procedure in core outcome set development: rating scale and consensus criteria determined outcome selection. J Clin Epidemiol. (2019) 111:30922885:23–31. doi: 10.1016/j.jclinepi.2019.03.011 30922885

[25] Dhas BN Alhadi S Al That G Al Abdulla SSH. Psychometric properties of the Arabic occupational balance questionnaire (OBQ11-a). Ann Med. (2024) 56:2346945. doi: 10.1080/07853890.2024.2346945, 38677318 PMC11057472

[26] Creek J. A standard terminology for occupational therapy. Br J Occup Ther. (2006) 69:202–8. doi: 10.1177/030802260606900502

[27] Brea Rivero M Creek J Meyer S Stadler-Grillmaier J Pitteljon H Faias J. Understanding the European conceptual framework for occupational therapy: for what it is worth. World Fed Occup Therap Bull. (2012) 65:12–9. doi: 10.1179/otb.2012.65.1.004

[28] Duncan EAS Murray J. The barriers and facilitators to routine outcome measurement by allied health professionals in practice: a systematic review. BMC Health Serv Res. (2012) 12:96. doi: 10.1186/1472-6963-12-96 22506982 PMC3358245

[29] Stapleton T McBrearty C. Use of standardised assessments and outcome measures among a sample of Irish occupational therapists working with adults with physical disabilities. Br J Occup Ther. (2009) 72:55–64. doi: 10.1177/030802260907200203

